# Usefulness of the newly developed artificial denture plaque for practical denture care training

**DOI:** 10.1002/cre2.270

**Published:** 2020-01-06

**Authors:** Katsuya Kawanishi, Tomoe Okahashi, Hideki Aita, Yuki Kan, Masao Yamazaki, Kenya Asahiro, Shizue Oyama, Takumi Ueki, Kana Sugihara, Riyo Chiba, Masa Yamagata, Kunio Matsubara, Yukie Murata, Kaname Shirai, Mai Kono, Mizuho Sasaki, Yoshifumi Toyoshita, Yasunori Sakakura, Toshiyuki Nagasawa, Hisashi Koshino

**Affiliations:** ^1^ Department of Occlusion and Removable Prosthodontics, School of Dentistry Health Sciences University of Hokkaido Tobetsu Japan; ^2^ Division of Advanced Clinical Education, Department of Integrated Dental Education, School of Dentistry Health Sciences University of Hokkaido Tobetsu Japan; ^3^ Dental Hygienist School Attached to School of Dentistry Health Sciences University of Hokkaido Tobetsu Japan; ^4^ Department of Geriatric Dentistry, Division of Human Biology and Pathophysiology, School of Dentistry Health Sciences University of Hokkaido Tobetsu Japan; ^5^ Dental Clinic Health Sciences University of Hokkaido Tobetsu Japan; ^6^ Department of Dental Hygiene, Faculty of Health Care Sciences Chiba Prefectural University of Health Sciences Chiba Japan

**Keywords:** clinical training, denture plaque control, educational tool, oral care

## Abstract

**Objectives:**

The aim of this study was to investigate whether the newly developed artificial dental plaque (A‐DP) is useful as an educational tool for denture care of dental hygienist that compared it with conventional artificial dental plaque from the viewpoint of practical skills.

**Material and methods:**

The 125 dental hygienist school students and 26 dental hygienists who had clinical experience were subjected a practical training of denture plaque control using the conventional denture plaque (C‐DP) and the A‐DP. The questionnaires based on the semantic differential method were used to survey whether the A‐DP is similar to the real denture plaque (R‐DP). Factor analysis by rotation of promax was carried out.

**Results:**

In the results of the factor analysis, the two factors could be detected in students and three factors in dental hygienists. The total score of each denture plaque was calculated for each factor, and correlation coefficient was examined. There was significant correlation between the A‐DP and the R‐DP at the first factors, both students and dental hygienists. C‐DP was not similar to R‐DP in all factors.

**Conclusions:**

These results suggested that A‐DP resembles R‐DP better than C‐DP. It was concluded that the A‐DP was similar to the R‐DP and could be a potent educational tool for practical denture care.

## 1 INTRODUCTION

Pneumonia had the third highest mortality rate by main cause of death in an overview of Japanese vital statistics from 2011 to 2016, and 90% of those deaths were in elderly people aged 65 years and older (Ministry of Health, Labor and Welfare Demographic Statistics, 2017). Reports indicate that more than approximately 70% of pneumonia cases in the elderly are aspiration pneumonia (Teramoto et al., 2008; Yamawaki, 2009). Focusing on the cause of death, ranking by sex and age, reveals that the mortality rate due to aspiration pneumonia has increased in elderly people aged 75 years and older (Ministry of Health, Labor and Welfare Demographic Statistics, 2017).

Plaque adhered to dentures not only causes oral diseases like denture stomatitis, angular cheilitis, root surface caries, and periodontitis but also causes aspiration pneumonia. Yoneyama, Yoshida, Matsui, and Sasaki (1999) reported that oral hygiene management for elderly people in need of long‐term care is an effective means of reducing fever and death due to pneumonia. Furthermore, the Guidelines for Treatment of Pneumonia in Adults (The Japanese Respiratory Society, 2017) has also reported the usefulness of oral hygiene management.

According to an international comparative report (Statistic Bureau, Ministry of Internal Affairs and Communications, 2018; United Nations, 2017) on the percentage of the total population aged 65 years or older in 2018, Japan had the highest percentage of elderly followed by Italy, Portugal, and Germany. The elderly population is also tending to increase in developed nations other than Japan, making oral hygiene management for the elderly an increasingly important issue.

Elderly people in need of long‐term care are unable to perform appropriate oral hygiene management or denture care by themselves, so they rely on nurses and long‐term care medical staff to perform this task (Stein & Henry, 2009). However, the dentures seen in nursing homes often still have food debris and denture plaque attached, so despite understanding the importance of oral hygiene management and denture care, there are still staff with insufficient knowledge and training (Costello & Coyne, 2008; Muramatsu & Moriya, 2014; Nishi, Mizuguchi, Nakamura, & Nagaoka, 2006; Young, Murray, & Thomson, 2008). Therefore, it is vital to secure human resources capable of implementing appropriate oral hygiene management. Thus, education material that reflects actual practices is absolutely essential to provide education on more practical and suitable denture care practices.

Practical denture training has been introduced into the oral hygiene management education in the dental hygienist college affiliated with this university, and the same training has been introduced for some of the students in the School of Nursing and Social Services, thereby ensuring coordinated education with other professions. However, the conventional artificial plaque® (Nissin Dental Products Inc., Kyoto, Japan) used for training is a manicure‐type material with an inorganic structure, so it has a different texture to real plaque. It is also sometimes dyed pink so that it can be seen as pigmentation depending on how it is viewed, and thus, it is difficult to say that it resembles real plaque found in the oral cavity. If this artificial plaque® is provisionally used as denture plaque for training, it will result in the same misconceptions. Moreover, it also poses the risk of deployment of practices focused on mechanical cleaning to remove “pigmentation assumed to be denture plaque.” Therefore, in the practical denture training to date, it has been necessary to explain these issues in advance to prevent these kinds of misconceptions. Because the artificial plaque® used for training has significantly deviated from the denture plaque encountered in actual clinical practice, in addition to tackling the development of denture plaque that more closely resembles the real thing, a great deal of consideration has been given to not only the color and texture but also the stainability and cleanability of the denture plaque to enable more realistic practical denture training. Thus, the following items have been set as requirements for artificial denture plaque for implementation in practical denture training.

・Have a texture and color tone specific to denture plaque.

・Be difficult to remove under running water.

・Be selectively stainable with disclosing solution.

・Enable objective evaluation of denture cleanliness with disclosing solution.

・Pose no physical risk to the student.

・Not affect the training environment.

・Have low environmental impact even when discarded.

The newly developed artificial denture plaque was examined to confirm whether this new type of artificial denture plaque reproduces the macroscopic and tactile characteristics of the real denture plaque encountered in clinical practice, and it was also compared with the artificial plaque® from the perspective of teaching the practical skills of plaque staining and denture cleaning.

In this study, we investigated whether the newly developed artificial denture plaque satisfies the requirements for realistic practical denture training and whether it is useful as an educational tool for denture cleaning instruction, and report our findings.

## 2 STUDY METHODS

### 2.1 Subjects

Practical denture training was implemented for 125 female students enrolled in the dental hygienist college affiliated with the School of Dentistry, Graduate School of Dentistry, Health Sciences University of Hokkaido (AY2015: 44 third‐year students, AY2016: 44 third‐year students, and AY2017: 37 third‐year students). The 26 dental hygienists who had more than 10 years of clinical experience also participated in this study.

### 2.2 Practical denture training material

#### 2.2.1 Conventional artificial denture plaque

**Figure 1:**
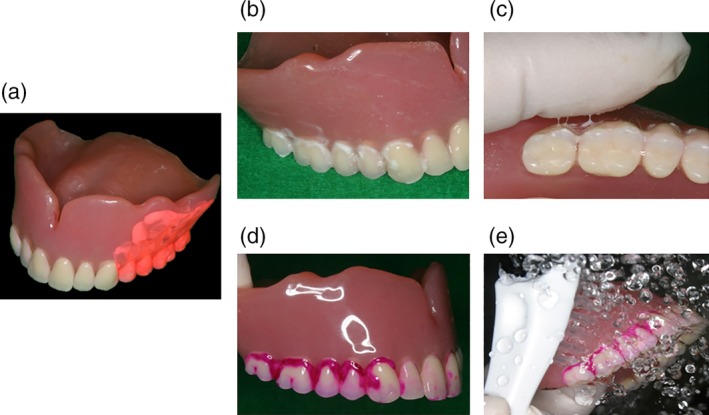
Artificial denture plaque adhered to the dentures. (a) Conventional artificial denture plaque adhered to the dentures. (b) Newly developed artificial denture plaque adhered to the dentures. (c) Macroscopic visual appearance and texture when palpated with fingers after the denture plaque adhered to the dentures. (d) The denture plaque after staining with disclosing solution. (e) The mechanical cleaning of the disclosed denture plaque

Artificial plaque® sold for brushing instruction was used for the conventional denture plaque (C‐DP; Figure 1a).

#### 2.2.2 Newly developed artificial denture plaque

Dental adhesive stabilizing agent (GlaxoSmithKline Consumer Healthcare Japan K.K, Tokyo, Japan.) mixed with ground chalk (Nihon Rikagaku Industry Co., Ltd, Kanagawa, Japan) at a dry‐weight ratio of 1:2 was used for the newly developed Artificial Denture Plaque (A‐DP; Figure 1b).

### 2.3 Artificial denture plaque evaluation method

A crossover trial was implemented with the students randomly assigned to two groups, one group starting practical denture training with C‐DP and the other group starting with A‐DP.

#### 2.3.1 Application of artificial denture plaque to the dentures

The students in each group attached the designated artificial denture plaque to the dentures in accordance with the following instructions. When using C‐DP, the plaque was applied evenly to the artificial tooth surface, the polished surface of the denture base, and the mucosal surface of the denture base and then left for 3 min to dry up naturally. When using A‐DP, it was applied after spraying distilled water onto the artificial tooth surface, the polished surface of the denture base, and the mucosal surface of the denture base. After shaking off the excess A‐DP, the surfaces were sprayed again with distilled water and left to dry up for 3 min (Figure 1b).

#### 2.3.2 Questionnaire evaluation using the semantic differential method

**Figure 2:**
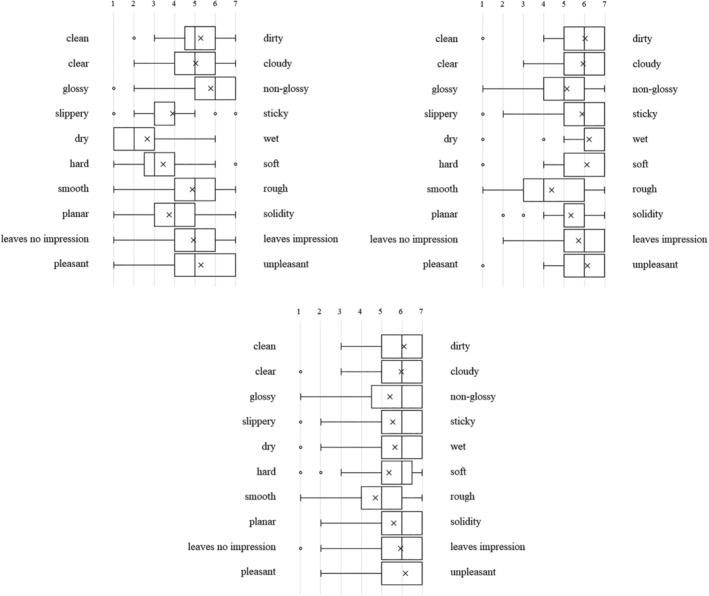
The macroscopic visual appearance and texture of the (a) C‐DP, (b) A‐DP, and (c) R‐DP adhered to the dentures when palpated with fingers

**Figure 3:**
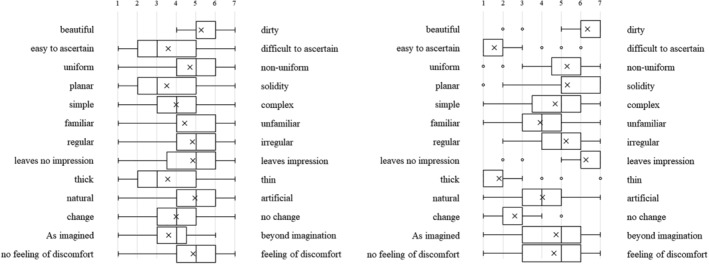
Visual assessment of the (a) C‐DP and (b) A‐DP after staining with disclosing solution

**Figure 4:**
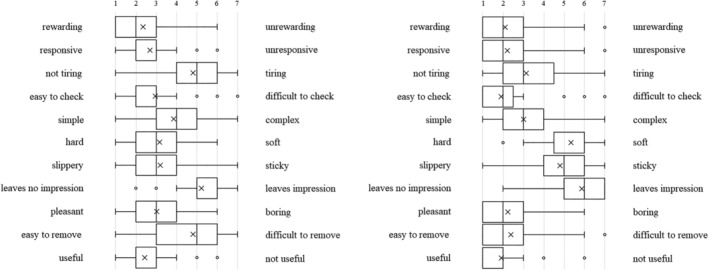
(a) Visual assessment of the A‐DP after staining with disclosing solution. (b) Assessment during mechanical cleaning of stained A‐DP

Each type of artificial denture plaque applied to the dentures was evaluated on the basis of macroscopic appearance, texture when palpated with fingers (Figure 1a–c), staining with disclosing solution (Figure 1d), and practical skills related to mechanical cleaning with a denture brush (Figure 1e) as well as using a questionnaire with a semantic differential method (SD method; Maki & Yamamoto, 1998). The content of the questionnaire was set as (1) evaluation of the denture plaque adhered to the dentures based on macroscopic visual appearance and texture when palpated with fingers, (2) visual assessment of the denture plaque after staining with disclosing solution, and (3) assessment during mechanical cleaning of the disclosed denture plaque. Each item was assessed using adjective pairs (Figures 2, 3, and 4). Finally, the dentures were immersed in the denture cleaning solution (GlaxoSmithKline Consumer Healthcare Japan K.K, Tokyo, Japan) for chemical cleaning.

Students who had experience with macroscopic checking and palpating real denture plaque (R‐DP) in actual clinical practice in a nursing care facility training or in dental clinics were asked to complete the questionnaire again for the questions in (1) above.

The dental hygienists evaluated both two types of artificial denture plaque adhered to the dentures and R‐DP and were asked to complete a questionnaire with an SD method.

#### 2.3.3 Questionnaire at completion of training

After completing this practical training, the students were asked to complete a questionnaire on whether they had experience with macroscopically checking an actual denture plaque, experience staining with disclosing solution, or experience with mechanical cleaning. They also completed a questionnaire composed of questions on their degree of interest in denture care.

### 2.4 Statistical analysis

The questionnaire results using the SD method of students and dental hygienists were collated for each question item, and factor analysis was implemented using the main factor method and promax rotation. After calculating the total score for each factor of C‐DP, A‐DP, and R‐DP, the correlation between corresponding factors of C‐DP, A‐DP, and R‐DP was investigated using Pearson's correlation coefficient.

The level of significance was set as a risk ratio of less than 5%, and IBM SPSS Statistics Ver 26.0 was used for all statistical analysis.

### 2.5 Ethical considerations

This study was approved by the School of Dentistry, Graduate School of Dentistry, Health Sciences University of Hokkaido, and the ethics committee of the School of Dentistry, Graduate School of Dentistry (Approval number: 119).

## 3 RESULTS

Of the 114 students remaining after excluding 11 students with incomplete responses to questionnaires using the SD method and the questionnaire after completion training, the results of the questionnaires were analyzed from 85 students with experience macroscopically checking and palpating R‐DP. In addition, we analyzed the correlation between the results of the questionnaires in 26 dental hygienists.

### 3.1 Results of each questionnaire using the SD method

**Figure 5:**
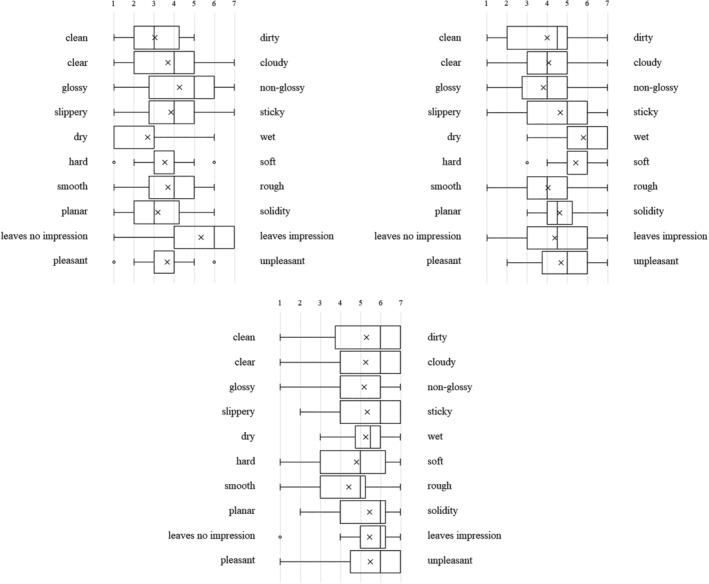
(a) The macroscopic visual appearance and texture of the (a) C‐DP, (b) A‐DP, and (c) R‐DP adhered to the dentures when palpated with fingers in dental hygienists

Figure 2 shows the SD profile of question items relating to the macroscopic visual appearance and texture of the denture plaque adhered to the dentures when palpated with fingers in students. When A‐DP (Figure 2b) and R‐DP (Figure 2c) were compared, it was found that both had almost the same points. On the other hand, C‐DP (Figure 2a) had points of difference with A‐DP and R‐DP in the multiple adjective pairs for both students and dental hygienists (Figure 5a,b). The SD profile of R‐DP (Figure 2c) showed a similar tendency to R‐DP (Figure 5c) in dental hygienists.

**Figure 6:**
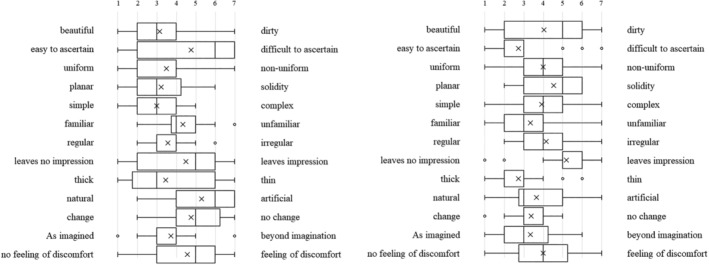
Visual assessment of the (a) C‐DP and (b) A‐DP after staining with disclosing solution in dental hygienists

Figure 3 shows the SD profile of question items relating to visual assessment of the denture plaque after staining with disclosing solution. When C‐DP (Figure 3a) and A‐DP (Figure 3b) were compared, it was found that both had similar points in the adjective pairs of “no feeling of discomfort‐feeling of discomfort,” for both students and dental hygienists (Figure 6a,b). Conversely, there were difference points in the multiple adjective pairs.

**Figure 7:**
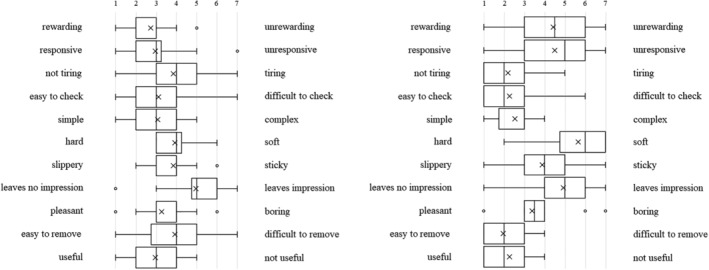
Assessment during mechanical cleaning of the stained (a) C‐DP and (b) A‐DP in dental hygienists

Figure 4 shows the SD profile of question items relating to assessment during mechanical cleaning of stained denture plaque. When C‐DP (Figure 4a) and A‐DP (Figure 4b) were compared, it was found that both had somewhat matching points in the adjective pairs of “rewarding‐unrewarding,” “responsive‐unresponsive,” “leaves no lasting impression‐leaves lasting impression,” and “useful‐not useful,” but the points differed for all remaining adjective pairs in the students. On the other hand, there were similar points in the adjective pairs of “slippery‐sticky,” “leaves no lasting impression‐leaves lasting impression,” and “pleasant‐boring,” in dental hygienists (Figure 7a,b).

### 3.2 Factor extraction and interpretation

The results of the R‐DP adjective pairs for questionnaires completed by students with experience in checking and palpating R‐DP were collated for each question item, and factor analysis was conducted using the main factor method and promax rotation to conduct a more detailed analysis of human perception. The results were divided into two factors in students (Table 1). The first factor collated the adjective pairs of “planar‐solidity,” “clean‐dirty,” “leaves no lasting impression‐leaves lasting impression,” “pleasant‐unpleasant,” “clear‐cloudy,” and “hard‐soft” and were called “visual and emotional characteristics.” The second factor collated the adjective pairs of “glossy‐non‐glossy,” “smooth‐rough,” and “slippery‐sticky” and were called “tactile characteristics.” On the other hand, The results were divided into three factors in dental hygienists (Table 2). The first factor was called “visual and emotional characteristics,” the second factor was called “visual and tactile characteristics,” and the third factor was called “tactile characteristics.”

**Table 1:** Factor extraction and interpretation

Question items		Factor loadings	
		I	II
Planar‐solidity		.801	−.126
Clean‐dirty		.777	.023
Leaves no impression‐leaves impression		.731	−.228
Pleasant‐unpleasant		.661	−.060
Dry‐wet		.646	.171
Clear‐cloudy		.611	.335
Hard‐soft		.572	.133
Glossy‐non‐glossy		−.199	.934
Smooth‐rough		−.071	.689
Slippery‐sticky		.177	.643
Factor	Correlation matrix between factors	I	II
Visual and emotional characteristics	I	‐	.527
Tactile characteristics	II		‐

**Table 2:** Factor extraction and interpretation in dental hygienists

Question items		Factor loadings		
		I	II	III
Pleasant‐unpleasant		.927	.041	−.107
Leaves no impression‐leaves impression		.737	−.355	.421
Clean‐dirty		.693	.308	−.004
Clear‐cloudy		.625	.432	−.070
Smooth‐rough		.511	.103	−.060
Planar‐solidity		−.186	.732	.295
Slippery‐sticky		.244	.620	−.096
Dry‐wet		.112	.464	−.033
Hard‐soft		−.111	.116	.869
Glossy‐non‐glossy		.215	.358	.394
Factor	Correlation matrix between factors	I	II	III
Visual and emotional characteristics	I	‐	.553	.358
Visual and tactile characteristics	II		‐	.256
Tactile characteristics	III			‐

The total points for C‐DP, A‐DP, and R‐DP, respectively, were calculated for each factor, and a significant, moderately strong correlation was found between A‐DP and R‐DP in the first factor (*r* = .475, *p* < .01) and a significant, weak correlation was found between A‐DP and C‐DP (*r* = .279, *p* < .05) as a result of examining Pearson's correlation coefficient in students (Table 3). A significant, strong correlation was found between A‐DP and R‐DP in the second factor (*r* = .636, *p* < .01) in students (Table 4).

**Table 3:** Result of examining Pearson's correlation coefficient in Factor 1

	C‐DP	A‐DP	R‐DP
C‐DP	‐		
A‐DP	.279*	‐	
R‐DP	.029	.475**	‐

* *p* < .05.

** *p* < .01.

**Table 4:** Result of examining Pearson's correlation coefficient in Factor 2

	C‐DP	A‐DP	R‐DP
C‐DP	‐		
A‐DP	.043	‐	
R‐DP	−.028	.636**	‐

** *p* < .01.

In dental hygienists, moderately strong correlation was found between A‐DP and R‐DP in the first factor (*r* = .446, *p* < .05), strong correlation was found between A‐DP and R‐DP in third factor (*r* = .647, *p* < .01), and the correlation was statistically significant (Tables 8 and 10).

### 3.3 Results of questionnaire after completion of training in students and dental hygienist

The results of the questionnaire after completion of training are shown in Table 5. Approximately half the students responded in the affirmative to the question “Have you ever stained real denture plaque with disclosing solution?” Approximately 90% of the students responded in the affirmative to the question “Have you ever mechanically cleaned real denture plaque?” Approximately 90% of the students wrote A‐DP in response to the question “Which denture plaque do you think would be effective to use for future instruction?” The first semester of second year was the most common response to the question relating to “Timing of practical denture training” (Table 6).

**Table 5:** The results of questionnaire after completion of training

Question items	Answer (numbers)	
	Yes	No
Have you ever stained real denture plaque with disclosing solution?	44	41
Have you ever mechanically cleaned real denture plaque?	80	5
Do you think that the content of the practical training will be useful in the future?	85	0
Did you participate positively in practical training?	85	0
Did you understand the content of the training?	85	0
Did you have any interest in denture care?	85	0
	ADP	C‐DP
Which denture plaque do you think would be effective to use for future instruction?	77	8

**Table 6:** Timing of practical denture training

Training stage		Students (numbers)
Grade	Semester	
First	First	1
	Last	4
Second	First	37
	Last	12
Third	First	15
	Last	16

The results extracting part of the comments section are shown in Table 7. In the interview to dental hygienist, a few dental hygienist mentioned that the artificial denture plaque required real the contents such as smell and color for training.

**Table 7:** The results extracting part of the comments section

・The denture plaque was truly realistic. ・The cleaning dentures was enjoyable. ・The training helped me understand the correct cleaning method. ・It was good to gain an interest in denture care. ・I was able to check the areas where dentures are prone to a build‐up of plaque. ・I have not had many opportunities to encounter denture plaque, so it was good to be able to understand the feeling of it. ・C‐DP is less like plaque, which made it difficult to get a true image of real plaque. ・A‐DP was more realistic than the C‐DP used previously, so it seemed more practical. ・A‐DP is sticky and resembles real denture plaque. ・The disclosing solution was not clearly apparent. ・How about overlaying A‐DP on C‐DP.

## 4 DISCUSSION

Dentists and dental hygienists have increased opportunities to conduct oral care and denture care in medical practice in Japan's super‐aging society, and dental care professions must communicate the correct knowledge and techniques to long‐term care medical staff. However, the reality is that students at dental hygienist schools have not attained a level of oral care that can be utilized in clinical practice (Aihara et al., 2011).

Thus, in this study, we devised the newly developed artificial denture plaque and evaluated whether this newly developed artificial denture plaque reproduces the macroscopic and tactile characteristics of real denture plaque encountered in clinical practice, using an SD method questionnaire to assess practical skills. We also compared the newly developed artificial denture plaque with conventional artificial denture plaque to determine its utility as an educational tool.

### 4.1 Devising the newly developed artificial denture plaque

Previously in the practical denture training in the dental hygienist college affiliated with this university, the artificial plaque® used for teaching brushing instruction was treated as denture plaque through application to the entire denture, and practical denture training mainly revolved around mechanical cleaning. Artificial plaque® appears as a pink color, so applying the plaque to a dental training model would stain that area pink, so when the color that was “assumed to be the color of plaque” adhered to the teeth, it could be recognized on a macroscopic level. Therefore, it was used as a useful educational tool for practicing mechanical cleaning methods using tooth surface cleaning equipment. However, real denture plaque encountered in clinical practice actually appears as a milky white color that is difficult to distinguish macroscopically from the color tone of artificial teeth. Elderly people and caregivers find it difficult to macroscopically differentiate this denture plaque from the dentures themselves, so using disclosing solution to demonstrate denture plaque during denture care training is considered an effective means of making the person aware of the adherence of plaque (da Silva & Paranhos Hde, 2006). Then, as a result of searching for material that could be stained with disclosing solution, we devised a plan to make a composite of commercially available dustless chalk®, as the color tone resembles the milky white color of denture plaque and its properties enable it to be stained with disclosing solution.

Furthermore, we determined that dental adhesive stabilizing agent was optimal for reproducing the specific texture of denture plaque given its similarity to viscous saliva, and its high adhesiveness in that it does not easily peel off under running water when washing dentures. Therefore, this was adopted as the material for the newly developed artificial denture plaque.

Methods for cleaning denture plaque can be divided into two types: mechanical cleaning and chemical cleaning (Felton et al., 2011). In mechanical cleaning, dentures are cleaned using a denture brush, and in chemical cleaning, the dentures are cleaned with denture cleanser. Mechanical cleaning is the main method used in training using conventional artificial denture plaque, but this method can damage the surface of the denture base (Kosuru et al., 2017). Therefore, the adhesiveness of the newly developed artificial denture plaque needed to be at a level that could easily be removed with soft brushing pressure, and dental adhesive stabilizing agent demonstrated an adequate adhesion force.

The dental adhesive stabilizing agent used in this study can be applied inside the oral cavity, so it is unlikely to cause problems for the students. The main component of dustless chalk® is scallop shells, so even if this material flows from the drain into waste water when washing the dentures with running water, the environmental pollution impact would be extremely small. This material may temporarily block drains, so it is necessary to attach a drain strainer as a preventative measure. Thus, we have fully considered the impact on the environment associated with practical denture training.

Denture plaque has also been reported as a cause of halitosis, but odor components were not added to the newly developed artificial denture plaque. The reason for this is that these components may have a negative impact on the training environment and objective evaluation of the condition of denture cleaning using disclosing solution is important, whereas evaluation of odor in clinical practice would be no more than a subjective evaluation. Although the dental hygienist who had clinical experience tend to seek real the contents such as smell and color, there are not necessarily to introduce in artificial denture plaque for training.

In this way, the raw materials used for the newly developed artificial denture plaque are comparatively inexpensive, familiar, and readily available, so they generate little economic burden and could be used for clinical training by long‐term care medical staff other than dental personnel, as they can be easily mixed by anyone.

### 4.2 Evaluation of the students and dental hygienists impression of each denture plaque using the SD method

The evaluation was conducted using the SD method to enable a multifaceted assessment of the visual and tactile image of the denture plaque adhered to the dentures, the image when using the disclosing solution, and the image when cleaning the dentures. The SD method is a method that can perform multidimensional evaluation of a single subject by combining multiple rating scales placed at either end of semantically conflicting adjectives. The obtained data can be summarized simply, and the mean value of each evaluation item can be connected with a line to create an SD profile. In this study, we adopted the most general 7‐point scale, and the seven points of “Extremely,” “Quite,” “Somewhat,” “Cannot say either way,” “Somewhat,” “Quite,” and “Extremely” were set for the adjective pairs.

Any student with no previous experience with macroscopically observing or touching denture plaque was unable to appropriately evaluate his or her impression of R‐DP and was therefore excluded from analysis.

The SD profile of R‐DP adhered to dentures presents a tendency biased towards a negative image in the adjective‐pairs of “clean‐dirty,” “clear‐cloudy,” and “pleasant‐unpleasant” (Figure 2c). A‐DP had a similar SD profile to R‐DP, demonstrating that the solidity and cloudiness characteristics specific to R‐DP were reproduced in the A‐DP. On the other hand, the image of adjective pairs for C‐DP, “slippery‐sticky,” “hard‐soft,” and “dry‐wet” differed significantly from the image of R‐DP. These results can be interpreted as indicating that the C‐DP observed by students during training diverged in part from the R‐DP encountered in clinical practice. The evaluation of R‐DP SD profile in dental hygienist showed a trend similar to R‐DP results of students (Figure 5c).

A‐DP SD profiles after staining the denture plaque were not similar to C‐DP SD profiles. A‐DP has a trend more strongly polarized distribution than C‐DP in students (Figure 3a,b). This is thought to be caused by C‐DP not being stained by the disclosing solution, whereas A‐DP had the characteristic of specifically staining only in the adhered areas.

C‐DP SD profiles during mechanical cleaning of denture plaque had a strong image of “hard,” “difficult to remove,” and “tiring” than A‐DP (Figure 4a,b). The results of dental hygienist also showed that A‐DP had a strong image of “easy to remove” compared with C‐DP (Figure 7a,b). This result suggests that C‐DP adheres strongly to dentures, whereas A‐DP has inferior adhesive force than C‐DP due to using a water soluble dental adhesive stabilizing agent. It was indicated that there is divergence point in the perceptions of both materials.

### 4.3 Factor analysis of R‐DP and comparison with each type of denture plaque

After receiving the results of the student impression evaluation using the SD method, the questionnaire results of the 85 students with experience observing or palpating R‐DP were collated, and factor analysis was conducted using the main factor method and promax rotation to analyze human perception in more detail (Table 1). Two factors were extracted as a result of factor analysis. A moderately positive inter‐factor correlation was found, and we found that the students' impression evaluation of R‐DP was interrelated. On the other hand, the dental hygienist' impression evaluation of R‐DP was extracted three factors and showed a moderately positive inter‐factor correlation.

We successfully interpreted the students' and dental hygienists' impression evaluation of R‐DP, so then, Pearson's correlation coefficient was used to investigate the correlation between A‐DP proposed in this study, R‐DP, and C‐DP.

In Factor 1, there was a significant, high correlation between R‐DP and A‐DP, indicating that A‐DP was similar to R‐DP in terms of “visual and emotional characteristics” in students (Table 3) and dental hygienists (Table 8). On the other hand, no correlation was found between C‐DP and R‐DP, indicating that C‐DP diverged from the denture plaque encountered in clinical practice (Table 9). Furthermore, C‐DP weakly correlated with A‐DP, indicating that both materials were recognized as artificial deposits in terms of “visual and emotional characteristics” (Tables 3 and 8).

**Table 8:** Result of examining Pearson's correlation coefficient in Factor 1 in dental hygienists

	C‐DP	A‐DP	R‐DP
C‐DP	‐		
A‐DP	.434*	‐	
R‐DP	.223	.446*	‐

* *p* < .05.

**Table 9:** Result of examining Pearson's correlation coefficient in Factor 2 in dental hygienists

	C‐DP	A‐DP	R‐DP
C‐DP	‐		
A‐DP	−.171	‐	
R‐DP	−.223	.337	‐

In Factor 2 in students and Factor 3 in dental hygienists, there was significantly high correlation between R‐DP and A‐DP, indicating that A‐DP had similar “tactile characteristics” to R‐DP (Tables 4 and 10). C‐DP was not similar to R‐DP in all factors, both students and dental hygienists. These results suggested that A‐DP resembles R‐DP better than C‐DP.

**Table 10:** Result of examining Pearson's correlation coefficient in Factor 3 in dental hygienists

	C‐DP	A‐DP	R‐DP
C‐DP	‐		
A‐DP	−.107	‐	
R‐DP	.196	.647**	‐

** *p* < .01.

### 4.4 Results of questionnaire after completion of training in students

There were many comments about an improved degree of interest and understanding of denture care in the free comments section of the questionnaire at the completion of training. Introduction of A‐DP in this training is thought to have contributed significantly to this outcome. A result that backs up this finding is that approximately 90% of the students selected A‐DP in response to the question “Which denture plaque do you think would be effective to use for future instruction?” A‐DP was evaluated highly, including comments like “A‐DP is sticky and resembles real denture plaque” and “I have not had many opportunities to encounter denture plaque, so it was good to be able to understand the feeling of it.” However, C‐DP was poorly evaluated, with comments like “C‐DP is less like plaque, which made it difficult to get a true image of real plaque” and “A‐DP was more realistic than the C‐DP used previously, so it seemed more practical.” Based on these results, introducing A‐DP into denture care led to an improved degree of interest and positivity towards denture care and improved the students' enthusiasm for learning.

On the other hand, there were also comments that the “disclosing solution was not clearly apparent.” This may have been caused by the ground chalk being washed off in flowing water or the dental adhesive stabilizing agent coating the chalk in an oblate shape. The dental adhesive stabilizing agent used in this study is water soluble, so it could be washed off under strong flowing water. However, when using material that adheres to the base of the dentures like conventional artificial denture plaque, applying brushing pressure with excessive force during mechanical cleaning is also a concern, so when conducting practical denture training, the current adhesive force and hardness of A‐DP should not present any problems. Among these comments, there was a suggestion to “try overlaying A‐DP on C‐DP,” so there were some students who were already aware of educational instruction and had developed a vision for instruction.

Many of the students expressed a preference for starting clinical training in the first semester of second year in response to the question on the timing of introducing practical denture training. There were also a large number of students who wished to utilize the knowledge and skills acquired in practical denture training in clinical practice.

### 4.5 Application to future training

Many of the areas where an undercut exists, like those found at the inner surface of the denture base and at the boundary between the denture and remaining teeth, have low levels of self‐cleaning, so denture plaque tends to adhere to these areas, and they tend to not be fully cleaned. The mucosal surface of maxillary tuberosity and the incisive papilla are reported to have a particularly high plaque adhesion rate (Fukuda, Yamauchi, Ogawa, Kim, & Tanahashi, 1996). Deliberately applying A‐DP to these common adherence sites and supplying these to students in a blinded state will reveal these areas for the first time to students when dyed with disclosing solution, which can then be used for educational instruction of the areas that tend to be overlooked, and to explain the significance of mechanical cleaning. It could also be used for evaluating residual denture plaque, revealed with disclosing solution, as an evaluation after denture cleaning. Furthermore, its use needs not be limited to dentures; A‐DP could also be applied to the teeth, mucosa, and tongue of jaw cast mannequins used for student practice, thereby also applying this technique to oral care cleaning instruction. A‐DP could be used not only for educating dental students and dental hygienist students but also for training nurses and long‐term care staff.

## 5 CONCLUSIONS

The differences between the newly developed artificial denture plaque proposed in this study and the conventional artificial denture plaque were evaluated with a questionnaire using the SD method via practical denture training. The results demonstrated that the newly developed artificial denture plaque resembles real denture plaque, and it satisfies the requirements to implement practical denture training. Therefore, it was found to be an effective educational tool for increasing the awareness of denture plaque in personnel involved in denture care and for providing practical instruction on cleaning methods.

## CONFLICT OF INTEREST

All authors declare that there is no conflict of interest.
